# Biological features and quality comprehensive analysis of twelve germplasm resources of the genus *Allium* from Tibet

**DOI:** 10.3389/fpls.2024.1393402

**Published:** 2024-08-06

**Authors:** Huaifeng Wang, Haixing Yang, Xuena Yu, Yongdong Xie, Yu Bai, Qiya Dai, Le Liang, Wen Tang, Mao Yong, Luzhou Wang, Zhi Huang, Bo Sun, Huanxiu Li, Yi Tang

**Affiliations:** ^1^ College of Horticulture, Sichuan Agricultural University, Chengdu, Sichuan, China; ^2^ Institute of Vegetables, Tibet Academy of Agricultural and Animal Husbandry Sciences, Lhasa, Tibet, China; ^3^ Institute of Agro-products Processing and Storage, Chengdu Academy of Agriculture and Forestry Sciences, Chengdu, Sichuan, China

**Keywords:** phenological period, quantitative characters, organosulfur compounds, amino acid, principal component analysis

## Abstract

**Introduction:**

*Allium* is important vegetables and seasonings in China, Tibet is rich in unique resources of the genus *Allium*, but lacks development and utilization.

**Methods:**

We compared the biological features and comprehensively evaluating the quality of twelve germplasm resources of the genus *Allium* collected from Tibet.

**Results:**

The results revealed that nine germplasm resources were bolting and bloom normally except for SC015, SC019, and SC048, all twelve germplasm resources were able to vegetative growth. The individual differences in moisture, soluble sugar, and protein content among the twelve germplasm resources were relatively small, with pyruvic acid content ranging from 0.11 to 1.12 mg/g and a large variation coefficient. A total of 8 categories and 97 volatile compounds were detected in twelve germplasm resources, the majority possessed the highest proportions of aldehydes and organosulfur compounds, but there were certain differences between the different *Allium* species. Additionally, 11 to 16 types of free amino acids were present in all germplasm resources, proline exhibited the highest content. The total content of essential and non-essential amino acids in SC009 was the highest. Carbon (C) accounted for the largest proportion of all elements, and the contents of other mineral elements varied greatly among the different plants.

**Conclusion:**

In conclusion, combined with biological performance and comprehensive evaluation of quality, SC009 is the excellent germplasm resource suitable for growth and capable of reproduction with good quality. These results improved the exploitation and utilization of the genus *Allium* in Tibet, as well as provided germplasm resources for high-quality breeding of the genus *Allium*.

## 1 Introduction

*Allium* L. is a perennial herb and belonging to the Amaryllidaceae family in the order Asparagales of monocotyledonous plants according to APG III (The Angiosperm Phylogeny Group, 2009) and APG IV (The Angiosperm Phylogeny Group, 2016). As the largest genera in the monocotyledon, the genus *Allium* contains 973 species and mainly distributed in tropical, temperate and semi-arid regions of the Northern Hemisphere (Sharifi-Rad et al., 2016; Hauenschild et al., 2017; Yan et al., 2023). Garlic (*A. sativum*), onion (*Allium cepa*), chive (*A. schoenoprasum*), shallot (*A. hirtifolium*), scallion (*A. chinense*), and leek (*Allium ampeloprasum*) are the most common and popular *Allium* members (Jin et al., 2018; Yan et al., 2023). These plants of genus *Allium* show rich biological activities including antimicrobial, antibacterial, antifungal, antiviral, antiparasitic, antioxidant, antitumor, immunoregulatory, and anti-inflammatory (Yan et al., 2023). For many years, they have been used as edible vegetable and medicinal to treat certain diseases such as fever, influenza, diabetes, arthritis, headache, hemorrhage, asthma, atherosclerosis, inflammatory diseases and other pathological condition (Lee et al., 2014; Marefati et al., 2021; Zolfaghari et al., 2021). Interestingly, it has recently been noted that these vegetables showed extensive potentials against the risk of cancer (Chu et al., 2017). The study on human leukocytes shows that onion extracts have strong protective effects on the DNA molecule and strong antiproliferative activity on human cancer cell lines, especially glioblastoma cells (Fredotović et al., 2017). The water leek extract at the concentration of 50 μg/mL suppresses the growth of MCF-7 cells human breast cancer cell more than 50% after incubation for three days (Zamri and Hamid, 2019). These functions, bioactivities, and health benefits are largely associated with the diverse bioactive components, including steroidal saponins, anthocyanins, gallic, ferulic acids, quercetin, alkaloids, and tannins (Asemani et al., 2019; Marefati et al., 2021). Furthermore, more and more studies suggest that much medical uses of the *Allium* species might be due to the presence of organosulfur compounds (OSCs), like ajoene, diallyl disulfide (DADS), diallyl trisulfide (DATS), polysulfanes, sulfoxides including allicin (S-allyll-L-cysteine sulfoxides), isoallicin (S-L-acryll-Lcysteine sulfoxides), methiin (S-methyl-L-cysteine sulfoxides) and propiin (S-propyl-L-cysteine sulfoxides) (Putnik et al., 2019; Subramanian et al., 2020). These organosulfur constituents not only possessed significant pharmacological activity, especially anticancer properties, but also give a sharp and tang odors and pungent spicy taste of *Allium* plants (Scherer et al., 2009; Alam et al., 2023). Because of abundant edible and medicinal value of *Allium* plants, they have been used as edible vegetable, medicinal and spice plant and widely cultivated worldwide (Scherer et al., 2009; Asemani et al., 2019; Bastaki et al., 2021).

At present, more than 141 million tons of crops of the genus *Allium* were produced worldwide (Yang et al., 2023), it has become the seventh largest vegetable that is cultivated and consumed throughout the world (Poojary et al., 2017; Fredotović and Puizina, 2019). The Qinghai-Tibet Plateau (QTP) is the highest and largest plateau in the world with an average elevation of greater than 4,000 m (Guo et al., 2022; Wang et al., 2023). Due to its vast territory, complex landforms, large differences in altitude, diverse climate, and ecological types, ecological diversity is extremely abundant with approximately 111 species of *Allium* genus growing on or surrounding the QTP (Hauenschild et al., 2017). Located in the core of the Qinghai-Tibet Plateau, Tibet is rich in unique resources of genus *Allium*, 32 species of wild resources have been discovered, including *A. atrosanguineum, A. hookeri, A. przewalskianum, A. fasciculatum*, and *A. sikkimense Baker* (Xie et al., 2019; Guo et al., 2022).

After a long-term scientific investigation, we collected a variety of cultivated and wild germplasm resources of the genus *Allium* in Tibet, but most of them have not been effectively developed and utilized. By comparing phenological phase, morphological characteristics, including plant height and fresh weight, leave number and size, pseudostem size, flowering stalk size, bud size, and comprehensively evaluating the basic nutrient quality and organosulfur compounds (OSCs), free amino acids, and mineral contents, we were intended to screen for the germplasm resource of the genus *Allium* that were suitable for growth and capable of reproduction with good quality. Our study not only effectively improves the exploration, development, and utilization of the resources of the genus *Allium* in Tibet, but also provides germplasm resources for high quality breeding of the genus *Allium*.

## 2 Materials and methods

### 2.1 Materials

Twelve germplasm resources of the genus *Allium* were collected from different regions of Tibet and uniformly planted in the nursery of the Tibetan Academy of Agricultural and Animal Husbandry Sciences. Detailed information of material number, name, type, and origin was provided in **Table 1**.

**Table 1:** Basic information of twelve germplasm resources of the genus *Allium*.

Material number	Species	Type	Altitude (m)	Origin
SC002	*Allium przewalskianum* Regel	Wild species	3548	Jiangzi County, Shigatse
SC004	*Allium przewalskianum* Regel	Wild species	3840	Luozha County, Shannan
SC007	*Allium przewalskianum* Regel	Wild species	4720	Gaize County, Ngari Prefecture
SC009	*Allium przewalskianum* Regel	Wild species	4630	Chamdo Prefecture
SC012	*Allium hookeri* Thwaites	Local species	2440	Nyingchi Prefecture
SC015	*Allium fistulosum* L. var. *viviparum Makino*	Local species	3260	Nyingchi Prefecture
SC019	*Allium przewalskianum* Regel	Wild species	4061	Cona County, Shannan
SC020	*Allium tuberosum* Rottl. ex Spreng	Local species	3111	Cona County, Shannan
SC021	*Allium hookeri* Thwaites	Local species	1707	Cona County, Shannan
SC022	*Allium tuberosum* Rottl. ex Spreng	Local species	3070	Cona County, Shannan
SC037	*Allium przewalskianum* Regel	Local species	3300	Lhunze County, Shannan
SC048	*Allium rhabdotum* Stearn	Wild relatives	4388	Lhunze County, Shannan

### 2.2 General situation of the test area

The test area is located in the Modern Agricultural Research and Development Base of Sichuan Agricultural University, Qiquan Town, Chongzhou City, Sichuan Province (N 30°33 ‘, E 103°39 ‘), with an altitude of 560 m above sea level. It exhibits a subtropical humid monsoon climate with an average annual temperature of 16°C, an average annual sunshine duration of 1,161.5 h, an average annual precipitation of 1,015.2 mm, and a frost-free period of 283 days a year. Snowfall is rare. The ground in the test area is flat, yellow loam with pH 6.76, organic matter content 31.68 g/kg, total nitrogen content 3.53 g/kg, total phosphorus content 0.32 g/kg, total potassium content 17.41 g/kg.

### 2.3 Sample collection and preparation

In March 2021 and March 2022, twelve germplasm resources of the genus *Allium* were transplanted to the experimental base, and the material planting was arranged according to random block method. The area of each experiment block was approximately 1.2 m^2^, and 30 plants of the same germplasm resources in each experiment block were planted with row and line spacing of 20 cm respectively, three repeated experiment blocks for per material, and consistent cultivation and management conditions of all experiment blocks. At different growth stages, ten plants in each experiment block were selected to observe phenological phases of each material in the year 2021 and 2022, and investigate quantitative characters of each material in the year 2022. Before bolting phase of each material in the year 2022, three representative plants were selected from each block, whole fresh plant was used to determine the moisture, the 2^nd^ or 3^rd^ mature leave from top to bottom was selected to determine the basic nutritional quality including soluble sugar, soluble protein, and pyruvic acid content, 17 kinds of free amino acids, the dried leave samples were used to determine mineral element content including carbon (C), nitrogen (N), potassium (K), sulfur (S), potassium (K), iron (Fe), manganese (Mn), zinc (Zn), and copper (Cu) content. Each material was repeated three times.

### 2.4 Observation of phenological phases

From March 2021 to October 2022, field observations of the phenological period were conducted on twelve germplasm resources of the genus *Allium* over two years. Ten plants from each material were labeled the dates of bolting, initial bloom, full bloom, final bloom, and seed maturity. The phenological phases described as follows. Bolting phase: the flower stem extends from the leaf sheath until the flower flocs break into buds; Initial bloom phase: the buds of 30% plants rupture in the plot; Full bloom phase: 90% of the plants bloom in the plot; Final bloom phase: 90% of the plants end of flowering. Seed maturity phase: 90% of the plant seeds mature (International Plant Genetic Resources Institute, 2001).

### 2.5 Investigation of quantitative characters

A total of 13 quantitative characters including plant height, tiller number, leaves number per plant, fresh weight per plant, leave length, leave width, pseudostem length, pseudostem diameter, flowering stalk length, flowering stalk diameter, bud length, bud width, and ball-flower diameter were investigated, ten plants were investigated for each germplasm resource and repeated three times. Besides, bud length and width were investigated before initial bloom phase, flowering stalk length and diameter, ball-flower diameter were investigated at full bloom phase, and other quantitative characters were investigated before bolting phase of each germplasm resource of the genus *Allium.* Plant height, leaf length, leaf width pseudostem length, flowering stalk length, bud length, and bud width were measured by millimeter scales, and plant height was determined from pseudostem base to uppermost part of plant, the 2^nd^ or 3^rd^ mature leave from top to bottom was selected to determine leaf length and leaf width. Pseudostem base, flowering stalk base, and the widest part of the bud was selected to measure pseudostem diameter, flowering stalk diameter, and ball-flower diameter using a vernier calipers. A balance was used to determine the fresh weight per plant (Hirata et al., 2016).

### 2.6 Determination of volatile compounds

Volatile compounds were determined using headspace solid-phase microextraction (HS-SPME) and gas chromatography/mass spectrometry (GC-MS). Clean and fresh leave samples (1 g) were transferred into a 15 mL headspace sample vial with a seal. Meanwhile, the extraction head was aged at 270°C at the inlet for 1h, then inserted into the top space of the sample bottle, and finally extracted in a 45°C water bath for 30 min. The extraction head was then inserted into the predesigned GC-MS (5979-6890N, Agilent Technologies Inc., Palo Alto, USA) injection port to thermally desorb volatiles in non-shunt mode. The determination conditions for GC-MS included a chromatographic column (DB-5MS: 30 m×250 μm×0.5 μm), an injection port temperature of 260°C, carrier gas (He), and a flow rate of 1.0 mL/min. For the programmed temperature rise, the temperature was maintained at 50°C for 2 minutes, increased to 150°C at 5°C/min, and finally increased to 250°C at 10°C/min, and then hold for 5 min. The injection volume was 1 μL. The spectral conditions included a bombardment ion source, and the ion source temperature was 230°C. The transmission line temperature was 280°C, and the electron energy was 70eV (Gao et al., 2021). The NIST Standards and Technology standard spectrum library was used to match the compounds, and the content of each component was calculated using the area normalization method.

### 2.7 Determination of basic nutritional quality

#### 2.7.1 Moisture content

Fresh overground part including pseudostem and leave was measured for fresh weight (Fw), and then dried at 80°C to constant weight to measure the dry weight (Dw). The calculation equation was as follows.

$\text{Moisture content}\left(\%\right)=\frac{\left(\text{Fw}-\text{Dw}\right)}{\text{Fw}}\times 100\%$ (Xu et al., 2023).

#### 2.7.2 Soluble sugar content

Frozen leave samples (0.5 g) were put into a test tube and added 5 ml distilled water, water bath at 85°C for 30 min, the supernatant was collected, and this step was repeated twice. Then the supernatant collected twice was filled to a volume of 10 ml with distilled water. The soluble sugar content was determined with the sulfuric acid anthrone method at a wavelength of 620 nm (Lin et al., 2013).

#### 2.7.3 Soluble protein content

Frozen leave samples (1.0 g) were ground up in a mortar with liquid nitrogen, 3 ml of phosphate-buffered solution (pH 7.0) was added to the mortar. Then the extract was centrifuged at 13,000×g for 15 min at 4 °C, and 0.1 ml of the supernatant was combined with 4.9 ml of Coomassie brilliant blue G-250 solution (0.1 g L^−1^). After 2 min, the soluble protein content was determined at a wavelength of 595 nm (Lin et al., 2013).

#### 2.7.4 Pyruvic acid content

Frozen leave samples (0.5 g) were ground with 10 mL trichloroacetic acid solution (8%) in an ice bath. After standing for 30 min, the mixture was centrifuged at 4000×g at 4°C for 10 min. Then 1 ml supernatant was transferred to another 10 ml test tube and added a mixed solution containing the following ingredients: 2 mL trichloroacetic acid (8%), 1 ml 2,4-dinitrophenylhydrazine (0.1%), and 5 ml NaOH (1.5 mol L^-1^). Then, the solution was shook for 10 min, and the pyruvic acid was determined at 520 nm (Gao et al., 2021).

### 2.8 Determination of free amino acid

The free amino acid content in the leave samples was determined using automatic amino acid analyzer (L-8900, Hitachi Co. Ltd., Tokyo, Japan) according to a previous report (He et al., 2018).

Preparation of standard solutions: 17 amino acid standards solutions containing Valine (Val), Methionine (Met), Isoleucine (Ile), Leucine (Leu), Phenylalanine (Phe), Histidine (His), Lysine (Lys), Threonine (Thr), Aspartate (Asp), Serine (Ser), Glutamate (Glu), Proline (Pro), Glycine (Gly), Alanine (Ala), Tyrosine (Tyr), Arginine (Arg), and Cystine (Cys) (>99%, Sigma, USA) was precisely diluted to 0, 1, 5, 10, 50 and 100 μmol/ml with 0.1 mol L^-1^ hydrochloric acid solution as calibration line.

Sample processing: 1 g of leave power was accurately weighed and placed in hydrolysis tubes, added 10 mL hydrochloric acid solution (6 mol/L), froze the samples for 5 min, vacuum-filled the samples with nitrogen gas three times, and finally sealed the samples in a nitrogen-filled state. After a sealed hydrolysis tube was placed in a 110°C constant temperature oven for hydrolysis for 22 hours, the hydrolysis solution was filtered into a 50 mL volumetric flask. The hydrolysis tube was rinsed multiple times and transferred to a volumetric flask at constant volume. After mixing, 1 mL of the filtrate was acquired and then concentrated and dried at 40°C. The residue was dissolved in 2 mL of water and condensed until it evaporated to dryness. A 2 mL aliquot of pH 2.2 sodium citrate buffer was added for dissolution. After shaking mixed, the sample was passed through a 0.22 μm filter membrane into the injection bottle.

Sample detection: the ninhydrin derivative of proline was monitored at 440 nm, and other amino acids were monitored at 570 nm. The contents of detected amino acids were quantified by comparing their peak areas with those of authentic standards.

Detection conditions: deamination column: 4.6mm×40 mm, #2650. Separation column: 4.6mm×60mm (packed with Hitachi custom ion exchange resin #2622, 3 μm), lithium type. Sample injection volume: 20 μL. Separation buffer: MCI Buffer L-8500-PF Kit (Mitsubishi Chemical Corporation). Flow rate: 0.35 mL min^−1^. Column temperature: 30-70°C. Reaction column: 4.6mm×40 mm, built-in inert silicon carbide particles. Reaction agent: Ninhydrin coloring solution kit for HITACHI. Reaction temperature: 135°C. Ninhydrin flow rate: 0.30 mL min^-1^.

### 2.9 Determination of mineral elements

Dried leave samples were crushed and sieved through a 100-mesh sieve. The total C content was determined using the potassium dichromate volumetric method (Yang and Yang, 2020). 0.03 g crushed leaf sample was weighed and placed in a test tube with. A standard solution of 0.8 mol L^-1^ potassium dichromate (5 mL) was added, followed by injection with 5 mL of concentrated sulfuric acid, after soaked for 24h, the digested solution was boiled at 245°C for 5min in a retort furnace. Then the mixture was quantified to 50 mL after cooling, and transferred to a 150 mL triangle bottle. Three drops of o-phenanthroline indicator were added to the bottle, and the mixture was titrated with a 0.2 mol L^-1^ FeSO_4_ standard solution. The end point occurred when the solution color changed from green to brownish red. The total carbon content of the sample was measured by titration volume.

Total N content in leave sample was assessed by the Kjeldahl method (El-Nakhel et al., 2020). 1.0 g of crushed leaf sample was placed in a glass tube with 7 mL of sulfuric acid (96%), 5 mL of hydrogen peroxide (30%) and 7 g of catalyzer containing selenium, potassium sulphate and copper oxide were added, then the tubes were placed for digestion in a heating plate for 30 min at 420 °C. Then the digested solution was distilled with the addition of 50 mL of 32% sodium hydroxide. Finally, the titration was conducted with sulfuric acid (0.1 mol L^-1^) in the presence of a colored indicator containing methyl red and bromocresol green, and the titration volume was used to calculate total nitrogen content. which was converted to g of nitrogen per kg of dry weight.

Crushed leaf sample (0.5 g) was accurately weighed and placed into a microwave digestion inner tank, followed by the addition of 10 mL of HNO_3_: HClO_4_ (4:1, V/V) mixed liquid, and the sample was digested overnight. After cooling, it was removed and degassed using ultrasound for 3 min, washed into a 50 mL volumetric flask, filtered through a 0.22 μm aqueous membrane (Gao et al., 2022). The sulfur (S), potassium (K), iron (Fe), manganese (Mn), zinc (Zn), and copper (Cu) contents were determined using an inductively coupled plasma-optical emission spectrometer (ICP-OES, iCAP-7400, Thermo Scientific, USA).

### 2.10 Statistical analyses

The (mean), standard deviation (SD), and coefficient of variation (CV) were calculated using Microsoft Excel 2017. IBM SPSS Statistics 21.0 software was used to analyze variance for all quantitative characters and biochemical traits using one-way ANOVA. Means were compared using Duncan multiple comparison at 5% significance levels. Origin 2021b was used for drawing column charts and finger-print, and the clustering and correlation analyses with an equidistant Pearson similarity analysis. TBtools was used to generate a heat map. Principal component analysis (PCA) was performed for factor analysis using SPSS.

## 3 Results

### 3.1 Phenological phase

There were significant differences in bolting and flowering times among the different germplasm resources. With the exception of SC015, SC019, and SC048 that were unable to bolt and bloom, the bolting and seed harvesting times of other germplasm resources were predominantly concentrated from May to August. SC002 exhibited the earliest bolting and flowering. SC004 possessed the earliest seed harvest time and shortest growth period with only 34 days from bolting to seed harvest. SC022 exhibited the latest bolting, flowering, and seed harvest times and the longest growth period with 73 days from bolting to seed harvest (**Table 2**).

**Table 2:** Phenological phases of twelve germplasm resources of the genus *Allium*.

Material number	Phenological phases (month.day)				
	Bolting phase	Initial bloom phase	Full bloom phase	Final bloom phase	Seed maturity phase
SC002	4.22	5.1	5.22	6.14	6.27
SC004	5.1	5.17	6.5	6.14	6.23
SC007	5.3	5.13	5.22	6.7	6.28
SC009	5.31	6.7	6.14	6.27	7.16
SC012	6.15	6.25	7.4	7.22	8.31
SC015	/	/	/	/	/
SC019	/	/	/	/	/
SC020	6.7	7.9	7.22	8.1	8.3
SC021	6.15	6.28	7.12	7.22	8.5
SC022	6.28	7.9	8.7	8.24	9.8
SC037	5.21	5.22	6.5	6.14	6.28
SC048	/	/	/	/	/

“/” represents the plant of genus Allium that did not bolt and blossom, and the data are not available.

### 3.2 Quantitative characteristics

**Figure 1:**
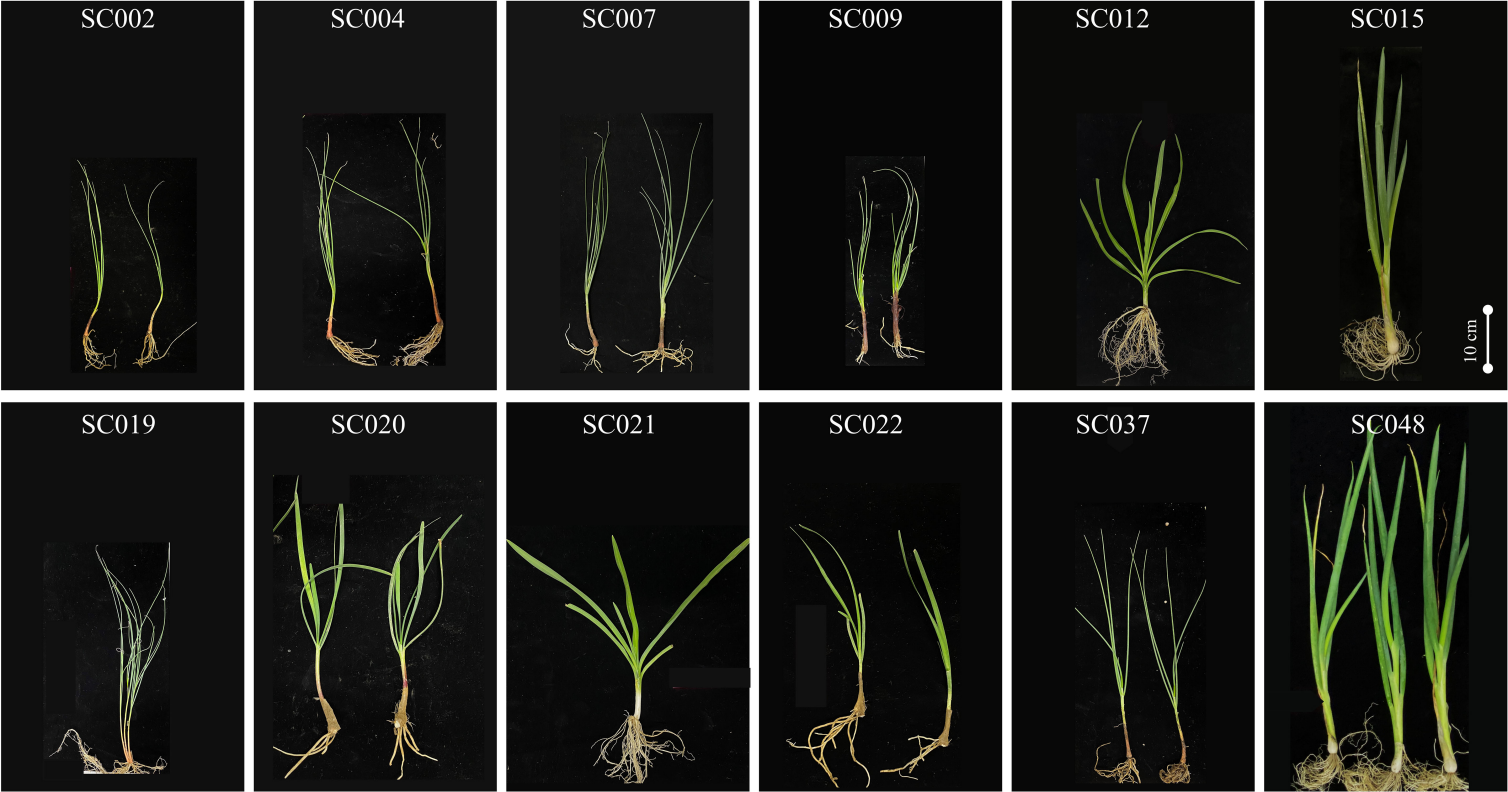
Appearance and morphology of twelve germplasm resources of the genus *Allium*.

The same quantitative characteristics exhibited significant differences among the different germplasm resources of the genus *Allium*, thus indicating high morphological diversity. The standard deviations of most quantitative traits were less than 10%, thus indicating high uniformity within the same species. The differences in plant height, leaf length, and pseudostem length among the different species were relatively small with low variation coefficient of 22.73%, 29.75%, and 33.91%, respectively. As the germplasms SC015, SC019, and SC048 were unable to bolt and bloom, the flowering stalk length, flowering stalk diameter, bud length, bud width, and ball-flower diameter were calculated to be zero. Therefore, the CV values of these five quantitative characteristics were greater than 67%. SC015 and SC048 possessed great advantages in terms of plant weight, and both were greater than 50 g. The CV of plant height was the highest and reached 135% (**Figure 1**; **Supplementary Table S1**).

### 3.3 Basic nutritional quality

**Figure 2:**
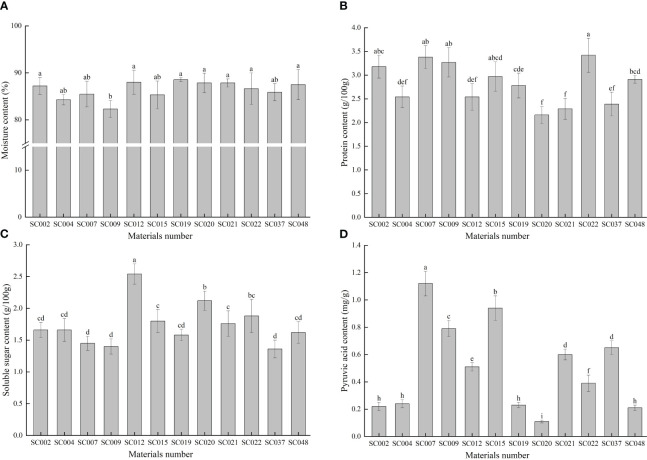
Comparison of basic nutritional qualities of twelve germplasm resources of the genus *Allium*. **(A)** Moisture content; **(B)** Protein content; **(C)** Soluble sugar content; **(D)** Pyruvic acid content.

The moisture content of all twelve germplasm resources of the genus *Allium* was higher than 80%, but the difference among the various plants was very small with a CV of only 2.12%. With the exception of SC009, there were no significant differences in moisture content among the other 11 germplasm resources (**Figure 2A**). The protein content in SC022 was the highest, but there was no significant difference compared to that of SC002, SC007, or SC009. The protein content of the different materials was relatively stable, and the maximum and minimum values did not exceed 25% of the average value (**Figure 2B**). The soluble sugar content was highest in SC012 and was significantly higher than that in the other materials, and it was the lowest in SC037. However, there was no significant difference compared to that of SC002, SC004, SC007, SC009, SC019, and SC048. The value was 21.39% lower than the average value (**Figure 2C**). The difference in pyruvic acid among the different germplasm resources was the largest with a CV of 64.89%. The highest content was in SC007 and was 10-fold higher than that of SC020 (**Figure 2D**).

### 3.4 Volatile compounds

**Figure 3:**
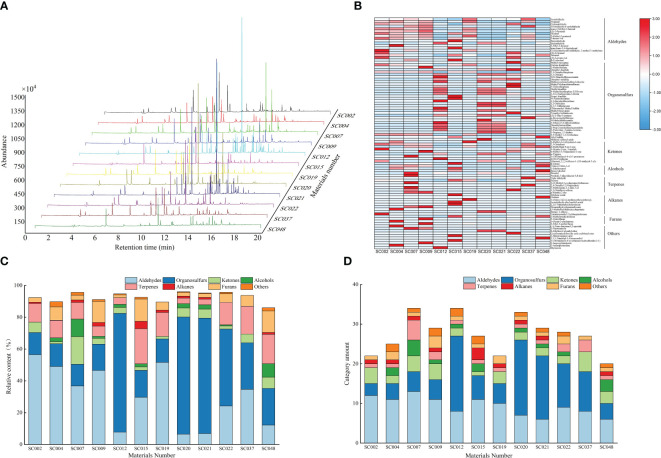
The volatile compounds in the twelve germplasm resources of the genus *Allium* as assessed by SPME-GC–MS. **(A)** Difference of the area of peaks for volatiles compounds; **(B)** Heat map of volatile compounds; **(C)** Relative content of different categories of volatile compounds; **(D)** Amount of each category of volatile compounds.

The volatile compounds in the leaves of twelve germplasm resources of the genus *Allium* were identified and analyzed using SPME-GC-MS. Based on parameters such as peak retention time and peak area, volatile compound maps of twelve germplasm resources were established, and the peak emergence time of all germplasm resources was concentrated between the 10^th^ and 16^th^ minutes (**Figure 3**). Eight types containing 97 kinds of volatile compounds were identified in the twelve germplasm resources, including 18 aldehydes, 33 sulfur-containing compounds, 10 ketones, 7 alcohols, 7 terpenoids, 9 alkanes, 4 furans, and 10 other compounds. Among them, crotonaldehyde, 2-hexenal, styrene, and 2-methyltetrahydrofuran were common compounds in the twelve germplasm resources with relative contents ranging from 9.06% to 48.45%. Acetaldehyde, propanal, 2-methyl-2-butenal, n-hexanal, 2-methyl-2-pentenal, benzaldehyde, nonanal, and 2,5-dimethylthiophene were common compounds in most plants (8 or more). These compounds were predominantly volatile aldehydes with a relative content ranging from 3.72% to 44.84% (**Supplementary Table S2**). A heat map was created based on the relative contents of volatile compounds. In general, the content of aldehyde compounds such as acetaldehyde, propanol, 2-methyl-2-butenal, and 2-methyl-2-pentenal in the six germplasm resources of *A. przewalskianum* (SC002, SC004, SC007, SC009, SC019, and SC037) was relatively high. The content of organic sulfur compounds such as dimethyl disulfide, 1,3-dithiane, and dimethyl trisulfide in the four germplasm resources of *A. hookeri* Thwaites and *A. tuberosum* Rottl. ex Spreng (SC012, SC020, SC021, and SC022) was higher than that in the other plants (**Figure 3B**).

The classification and statistics of volatile compounds in all germplasm resources of the genus *Allium* revealed that the total content of volatile compounds in all germplasm resources exceeded 80%, and the composition of volatile compounds was consistent. However, the contents were significantly different. With the exception of SC015 and SC048 that are two scallion plants, the volatile compounds in the other germplasm resources were primarily aldehydes and organosulfur, and the total relative contents of these two categories exceeded 50%. SC007 and SC012 possessed the most abundant volatile compounds (34 types), whereas SC048 possessed the least number of volatile compounds (20 types). There were 12 aldehyde compounds in SC002 with a relative content of 56.47% that accounted for the largest proportion, whereas seven aldehyde compounds in SC020 accounted for only 6.54%. There were 19 OSCs in SC012 and SC020 with contents of 74.68% and 73.59%, respectively, and these were the highest among all the plants. There were four ketone and four terpenoid compounds in SC007 with relative contents of 17.31% and 11.07%, respectively, and both of these were the highest. The relative content and types of alkane compounds were relatively low in all germplasm resources with a maximum of only 4.69% in SC015, and the three germplasm resources did not contain such compounds. A total of one to three types of furan compounds were detected in the different germplasm resources, and the highest relative content of 13.46% was observed in SC048 (**Figures 3C, D**).

### 3.5 Free amino acid

Sixteen free amino acids were detected in twelve germplasm resources of the genus *Allium*, including 8 essential amino acids (EAA) identified as Val, Met, Ile, Leu, Phe, His, Lys, and, Thr, and 8 non-essential amino acids (NEAA) identified as Asp, Ser, Glu, Pro, Gly, Ala, Tyr, Arg. In terms of EAA, the highest content was Lys was 0.18g/100 g in SC009, and this was significantly higher than that in other plants and 38.46% higher than the average value. Next was Leu that exhibited the highest content in both SC037 and SC048. Met content was the lowest among EAA with an average value of only 0.02 g/100 g, and Met was not detected in SC015 and SC048. There was little difference in the content of Val, Ile, Phe, Lys, and Thr among different germplasm resources, and the CV values were all less than 25%; As Met and His were detected in some germplasm resources, the CV values of these two EAA exceeded 55%. The total EAA content varied slightly among the different plants with a CV of 19.24%. In SC002, the total EAA content was the lowest (37.5% lower than the average), and it was the highest in SC009 at 41.67% higher than the average (**Table 3**). Pro content was significantly higher than that of the other NEAA, and the highest content was 1.61g/100 g in SC009. The Ser, Gly, and Tyr contents were relatively low with average values less than 0.1 g/100 g. The differences in Tyr and Arg content among the different germplasm resources were relatively small with a CV below 22%, and the difference in Ala content was the largest with a CV exceeding 100% (**Table 4**).

**Table 3:** Comparison of essential amino acids in twelve germplasm resources of the genus *Allium*.

Materials Number	Content of essential amino acid (g/100g)								
	Val	Met	Ile	Leu	Phe	His	Lys	Thr	Total
SC002	0.06 ± 0.00e	0.01 ± 0.00d	0.06 ± 0.00e	0.09 ± 0.01f	0.06 ± 0.00e	0.03 ± 0.00d	0.08 ± 0.01e	0.06 ± 0.01d	0.45 ± 0.04g
SC004	0.11 ± 0.01bc	0.01 ± 0.00d	0.10 ± 0.00b	0.02 ± 0.00g	0.11 ± 0.01b	0.04 ± 0.00b	0.13 ± 0.01c	0.10 ± 0.01c	0.61 ± 0.01f
SC007	0.11 ± 0.01bc	0.01 ± 0.00d	0.10 ± 0.01b	0.02 ± 0.00g	0.11 ± 0.01b	0.04 ± 0.00b	0.14 ± 0.01bc	0.10 ± 0.02c	0.63 ± 0.01ef
SC009	0.14 ± 0.01a	0.01 ± 0.00d	0.13 ± 0.01a	0.22 ± 0.02a	0.15 ± 0.01a	0.06 ± 0.01a	0.18 ± 0.01a	0.13 ± 0.01b	1.02 ± 0.06a
SC012	0.10 ± 0.00cd	0.03 ± 0.00b	0.08 ± 0.01d	0.15 ± 0.01bcd	0.10 ± 0.01bc	0.04 ± 0.00bc	0.13 ± 0.01c	0.09 ± 0.00c	0.72 ± 0.04bcd
SC015	0.09 ± 0.01d	/	0.08 ± 0.01d	0.13 ± 0.01de	0.08 ± 0.01cd	0.04 **±** 0.01bc	0.10 ± 0.01d	0.17 ± 0.02a	0.69 ± 0.06bcde
SC019	0.10 ± 0.01cd	0.02 ± 0.00c	0.09 ± 0.00bc	0.16 ± 0.01bc	0.11 ± 0.01b	0.04 ± 0.01bc	0.13 ± 0.01c	0.10 ± 0.01c	0.75 ± 0.04bc
SC020	0.08 ± 0.00d	0.02 ± 0.00c	0.07 ± 0.01d	0.12 ± 0.01e	0.08 ± 0.01d	0.03 ± 0.00cd	0.11 ± 0.01d	0.14 ± 0.01b	0.66 ± 0.05cde
SC021	0.10 ± 0.01cd	0.04 ± 0.01a	0.08 ± 0.01cd	0.15 ± 0.01bcd	0.09 ± 0.01cd	0.04 ± 0.00b	0.11 ± 0.01d	/	0.61 ± 0.06f
SC022	0.10 ± 0.01cd	0.03 ± 0.00b	0.08 ± 0.00d	0.15 ± 0.00cd	0.09 ± 0.00cd	0.04 ± 0.00bc	0.13 ± 0.01c	0.10 ± 0.01c	0.71 ± 0.03abcd
SC037	0.12 ± 0.01b	0.02 ± 0.00c	0.10 ± 0.01b	0.17 ± 0.02b	0.10 ± 0.02bc	0.04 ± 0.00b	0.15 ± 0.01b	/	0.71 ± 0.03abcd
SC048	0.11 ± 0.01bc	/	0.10 ± 0.01b	0.17 ± 0.01b	0.11 ± 0.01b	0.04 ± 0.00b	0.15 ± 0.01b	0.10 ± 0.01c	0.78 ± 0.06b
Average	0.12	0.02	0.09	0.13	0.10	0.04	0.13	0.09	0.69
CV (%)	18.55%	65.42%	20.29%	46.17%	22.75%	17.47%	19.93%	55.22%	19.24%

“/” represents not detected. Data represents means (± SD) of three independent replicates. Different lowercase letters indicate significant differences within a column (LSD P ≤ 0.05).

**Table 4:** Contents of non-essential amino acids in twelve germplasm resources of the genus *Allium*.

Materials Number	Content of non-essential amino acid (g/100g)								
	Asp	Ser	Glu	Pro	Gly	Ala	Tyr	Arg	Total
SC002	0.14 ± 0.01e	0.06 ± 0.01e	0.25 ± 0.03d	0.82 ± 0.11d	0.06 ± 0.01d	0.72 ± 0.05a	0.04 ± 0.00e	0.08 ± 0.00e	2.17 ± 0.20bc
SC004	0.22 ± 0.02c	0.10 ± 0.01cd	0.28 ± 0.02d	1.19 ± 0.17bc	0.11 ± 0.01b	0.12 ± 0.01cd	0.07 ± 0.01bc	0.13 ± 0.01bc	2.22 ± 0.23bc
SC007	0.20 ± 0.02cd	0.11 ± 0.01cd	0.29 ± 0.02cd	1.26 ± 0.15b	0.11 ± 0.01b	0.12 ± 0.01cd	0.08 ± 0.01b	0.13 ± 0.02bc	2.30 ± 0.21bc
SC009	0.31 ± 0.03 a	0.14 ± 0.02b	0.38 ± 0.02b	1.61 ± 0.18a	0.15 ± 0.01a	0.16 ± 0.01b	0.10 ± 0.01a	0.17 ± 0.01cd	3.01 ± 0.28a
SC012	0.18 ± 0.00d	0.10 ± 0.01cd	0.35 ± 0.04b	0.05 ± 0.00e	0.11 ± 0.01b	0.11 ± 0.01cd	0.07 ± 0.00bc	0.11 ± 0.01cd	1.09 ± 0.08e
SC015	/	/	0.13 ± 0.01e	/	/	0.10 ± 0.01cd	0.05 ± 0.00d	0.11 ± 0.01cd	0.39 ± 0.02f
SC019	0.19 ± 0.01d	0.10 ± 0.00cd	0.25 ± 0.01d	1.14 ± 0.02bc	0.11 ± 0.00b	0.12 ± 0.01cd	0.07 ± 0.01bc	0.12 ± 0.01bcd	2.10 ± 0.04c
SC020	/	0.07 ± 0.01e	0.24 ± 0.03e	1.04 ± 0.14c	0.08 ± 0.01c	0.09 ± 0.01d	0.05 ± 0.01d	0.10 ± 0.01d	1.67 ± 0.18d
SC021	/	0.16 ± 0.02a	0.12 ± 0.01d	/	0.12 ± 0.01b	/	0.06 ± 0.01cd	0.11 ± 0.01cd	0.57 ± 0.04f
SC022	0.22 ± 0.01	0.11 ± 0.00c	0.34 ± 0.02bc	0.05 ± 0.00e	0.11 ± 0.01b	0.11 ± 0.01cd	0.07 ± 0.01bc	0.12 ± 0.01bcd	1.13 ± 0.05e
SC037	0.25 ± 0.02b	0.09 ± 0.01d	0.45 ± 0.05a	1.25 ± 0.14b	0.11 ± 0.01b	0.13 ± 0.01c	0.08 ± 0.01b	0.14 ± 0.01b	2.50 ± 0.25b
SC048	0.18 ± 0.01d	0.11 ± 0.01cd	0.28 ± 0.03d	1.27 ± 0.15b	0.11 ± 0.02b	0.12 ± 0.01cd	0.08 ± 0.01b	0.14 ± 0.02b	2.29 ± 0.24bc
Average	0.16	0.10	0.28	0.81	0.10	0.14	0.07	0.12	1.79
CV (%)	63.93	42.22	34.14	74.81	37.53	104.58	21.88	19.26	45.51

“/” represents not detected. Data represents means (± SD) of three independent replicates. Different lowercase letters indicate significant differences within a column (LSD P ≤ 0.05).

### 3.6 Mineral elements

**Figure 4:**
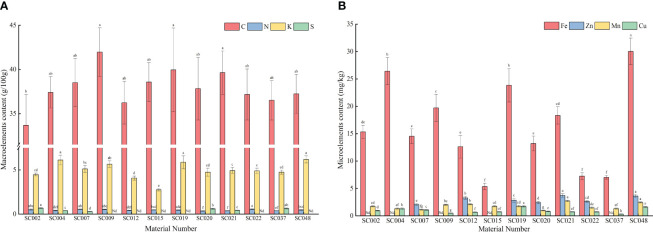
Contents of mineral elements in twelve germplasm resources of the genus *Allium*. **(A)** Macroelements content; **(B)** Microelements content. Note: “Nd” in the figure indicates that it is not detected.

Mineral elements were detected in the above-ground portions of twelve germplasm resources of the genus *Allium*. Among the macroelements, the C content in all germplasm resources were relatively high and ranged from 33.69 to 41.97 g/100 g. The difference between the different germplasm resources was small with the highest content in SC009. The only significantly different amount was that in SC002. The N content was the highest in SC002, but there was no significant difference compared to the content in SC002, SC007, and SC009 that was 57.34% higher than the lowest N content in SC020. The K content in SC004, SC019, and SC048 was significantly higher than that in the other eight germplasm resources with the exception of SC009. The K content in SC015 was the lowest with only 43.62% in SC048, and this was significantly lower than that of the other germplasm resources. The maximum difference in S content between different germplasm resources was only detected in SC002, SC004, SC007, SC020, SC021, and SC047, where the highest content was observed in SC002. This was not significantly different compared to that in SC037. No P content was detected in any of the plants, and therefore, the data are not listed (**Figure 4A**). In terms of microelements, SC048 possessed the highest Fe content, and this was followed by SC004 and SC019 and was significantly higher than that in the other germplasm resources. The Mn content was the highest in SC021, but there was no significant difference compared to the content in SC048 that was 2.78-fold higher than that of SC015. The Cu content in SC019 and SC048 was significantly higher than that in the other germplasm resources, whereas the Cu content in SC037 was the lowest at only 35.55% of the average value. The Zn content in SC021 and SC048 was significantly higher than that in the other germplasm resources, but due to the absence of Zn detection in SC002, SC004, SC009, SC015, and SC037, the Zn content varied the most among the twelve germplasm resources with a CV of up to 92.20% (**Figure 4B**).

### 3.7 Correlation analysis of quality

**Figure 5:**
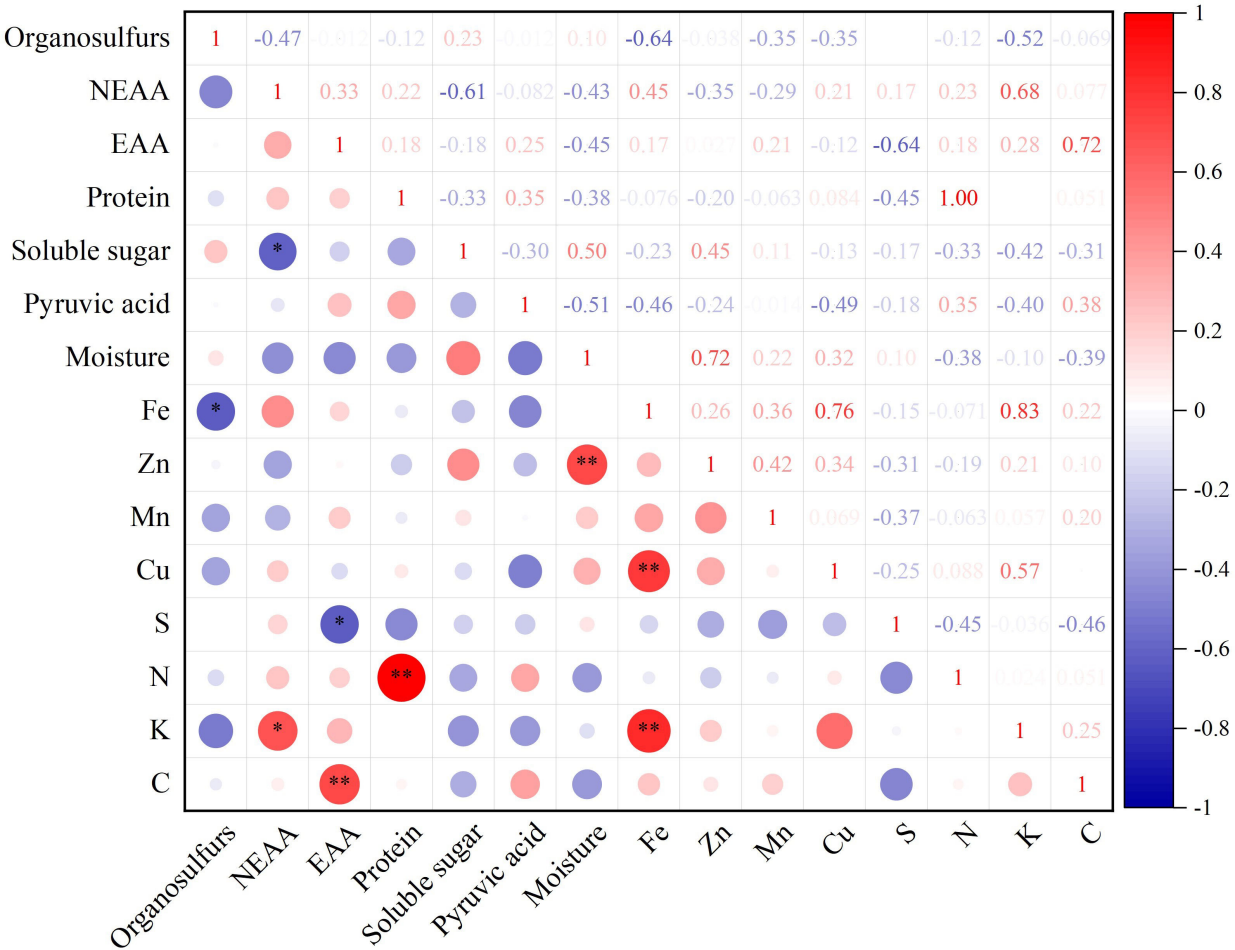
Quality correlation analysis of twelve germplasm resources of the genus *Allium*. * Correlation is significant at the 0.05 level, ** Correlation is significant at the 0.01 level.

Correlation analysis of 15 quality indices, including NEAA, EAA, protein, soluble sugar, pyruvic acid, moisture, Fe, Zn, Mn, Cu, S, N, K, and C content in different germplasm resources of the genus *Allium*, revealed a highly significant positive correlation between EAA and C, protein and N, Zn and moisture, and Fe and Cu and K content (*P*<0.01) with correlation coefficients of 0.72, 1.00, 0.72, 0.76, and 0.83, respectively. There was a significant negative correlation between organosulfur and Fe, NEAA, soluble sugar, EAA, and S contents (**Figure 5**).

### 3.8 Principal component analysis quality

Principal component analysis was conducted on 15 quality indicators of twelve germplasm resources of the genus *Allium*, and 5 principal components with eigenvalues of greater than 1 were extracted. The cumulative contribution of the 5 principal components was 85.37% (**Table 5**). According to the loading matrix, principal component 1 was jointly influenced by six indices that included NEAA, EAA, K, soluble sugar, and moisture. Among them, NEAA, EAA, and K possessed the highest positive loadings, whereas soluble sugars possessed the highest negative loadings. Based on the correlation analysis, NEAAs were positively correlated with K and negatively correlated with soluble sugar content. Therefore, principal component 1 mainly represented the amino acid composition factors of the twelve germplasm resources of the genus *Allium*. Principal component 2 was primarily influenced by Fe, Zn, Cu, K, and pyruvic acid content with pyruvic acid as a negative loading control, whereas principal component 3 was predominantly influenced by S, Zn, and Cu, with S exhibiting the highest absolute loading value. Therefore, principal components 2 and 3 primarily represent the essential element composition factors of the 12 *Allium* sources. The representative indices of principal component 4 were protein and N, and correlation analysis revealed a significant positive correlation between protein and N content. Therefore, principal component 4 represented the basic nutritional quality components that were primarily composed of proteins. The loading value of organic sulfur in principal component 5 was the highest, thus indicating that principal component 5 mainly reflected the sulfur-containing volatile components of germplasm resources of the genus *Allium* (**Table 6**).

**Table 5:** Eigenvalue and contribution rate of principal components.

Principal component	Characteristic value	Variance contribution rate	Cumulative contribution rate
1	3.988	26.585	26.585
2	3.515	23.436	50.021
3	2.504	16.691	66.712
4	1.719	11.459	78.172
5	1.08	7.201	85.372

**Table 6:** Principal component loading matrix of quality of twelve germplasm resources of the genus *Allium* .

Quality index	Principal component				
	1	2	3	4	5
Organsulfurs	-0.525	-0.414	0.136	-0.062	0.616
NEAA	0.722	0.096	-0.541	-0.085	0.113
EAA	0.592	-0.144	0.491	-0.444	0.282
Protein	0.557	-0.425	0.166	0.678	0.013
Soluble sugar	-0.686	0.187	0.398	0.089	0.104
Pyruvic acid	0.215	-0.732	0.252	-0.127	-0.365
Moisture	-0.618	0.596	0.171	0.260	0.017
Fe	0.556	0.785	-0.051	-0.052	-0.066
Zn	-0.213	0.615	0.576	0.091	0.047
Mn	0.077	0.360	0.590	-0.093	-0.577
Cu	0.326	0.719	-0.019	0.377	0.201
S	-0.379	0.017	-0.830	-0.181	-0.212
N	0.560	-0.419	0.163	0.679	0.014
K	0.657	0.620	-0.213	-0.109	0.160
C	0.523	-0.084	0.477	-0.540	0.116

### 3.9 Comprehensive evaluation of quality

A comprehensive quality evaluation of twelve germplasm resources of the genus *Allium* was conducted.

$\text{Factor eigenvector coefficient}=\frac{\text{Principal component loading}}{\sqrt{\text{Corresponding principal component eigenvalues}}}$ .

After calculating the eigenvector coefficients of each principal component, the score function of 1-5 principal components was constructed as follows:

y_1_=–0.26X_1_+0.36X_2_+0.30X_3_+0.28X_4_–0.34X_5_+0.11X_6_–0.31X_7_+0.28X_8_–0.11X_9_+0.04X_10_+0.16X_11_–0.19X_12_+0.28X_13_+0.33X_14_+0.26X_15_

y_2_=–0.22X_1_+0.05X_2_–0.08X_3_–0.23X_4_+0.10X_5_–0.039X_6_+0.32X_7_+0.42X_8_+0.33X_9_+0.19X_10_ +0.39X_11_+0.01X_12_–0.22X_13_+0.33X_14_–0.04X_15_

y_3_ = 0.08X_1_–0.34X_2_+0.31X_3_+0.11X_4_+0.25X_5_+0.16X_6_+0.11X_7_–0.03X_8_+0.36X_9_+0.37X_10_–0.01X_11_–0.52X_12_+0.10X_13_–0.13X_14_+0.30X_15_

y_4_=–0.05X_1_–0.06X_2_–0.34X_3_+0.52X_4_+0.07X_5_–0.10X_6_+0.20X_7_–0.04X_8_+0.07X_9_–0.07X_10_ +0.29X_11_–0.14X_12_+0.52X_13_–0.08X_14_–0.41X_15_

y_5_ = 0.59X_1_+0.11X_2_+0.27X_3_+0.01X_4_+0.10X_5_–0.35X_6_+0.02X_7_–0.06X_8_+0.05X_9_–0.56X_10_ +0.19X_11_–0.20X_12_+0.01X_13_+0.15X_14_+0.11X_15_

where y1, y2, y3, y4, and y5 are the eigenvector weights of one to five principal components, and X1, X2,…, and X15 are the normalized values of organosulfur, NEAA, EAA, protein, soluble sugar, pyruvate, water, Fe, Zn, Mn, Cu, S, N, K, and C contents for each *Allium* plant. Comprehensive quality score of *Allium* plants, $\text{y}=\frac{\text{t}1}{\text{t}1+\text{t}2+\text{t}3+\text{t}4+\text{t}5}\times \text{y}1+\frac{\text{t}2}{\text{t}1+\text{t}2+\text{t}3+\text{t}4+\text{t}5}\times \text{y}2+\frac{\text{t}3}{\text{t}1+\text{t}2+\text{t}3+\text{t}4+\text{t}5}\times \text{y}3+\frac{\text{t}4}{\text{t}1+\text{t}2+\text{t}3+\text{t}4+\text{t}5}\times \text{y}4+\frac{\text{t}5}{\text{t}1+\text{t}2+\text{t}3+\text{t}4+\text{t}5}\times \text{y}5$

That is, y=0.31y1 + 0.27y2 + 0.20y3 + 0.13y4 + 0.08y5, where t1, t2, t3, t4, and t5 were the eigenvalues of the 1 to 5 principal components, respectively (Han et al., 2022). The comprehensive scores from highest to lowest were SC048, SC019, SC009, SC004, SC007, SC022, SC012, SC021, SC002, SC020, SC015, and SC037 (**Table 7**).

**Table 7:** Score and sorting of principal component factors of quality of twelve germplasm resources of the genus *Allium*.

Material number	y1	y2	y3	y4	y5	Synthesis score	Sort
SC048	0.50	0.78	0.18	0.09	0.02	1.58	1
SC019	0.41	0.67	0.13	0.03	0.06	1.31	2
SC009	1.31	–0.52	0.16	–0.21	–0.01	0.73	3
SC004	0.33	0.36	–0.42	–0.07	0.02	0.22	4
SC007	0.53	–0.37	–0.09	0.16	–0.04	0.19	5
SC022	–0.16	–0.38	0.24	0.23	0.12	0.04	6
SC012	–0.67	0.18	0.37	0.02	–0.06	–0.16	7
SC021	–0.47	0.38	0.25	–0.19	–0.16	–0.20	8
SC002	–0.23	0.00	–0.48	0.30	–0.10	–0.51	9
SC020	–0.84	0.20	–0.20	–0.17	0.15	–0.85	10
SC015	–0.46	–0.86	0.29	0.03	0.01	–0.99	11
SC037	–0.24	–0.45	–0.42	–0.22	–0.01	–1.34	12

### 3.10 Clustering analysis of quality

**Figure 6:**
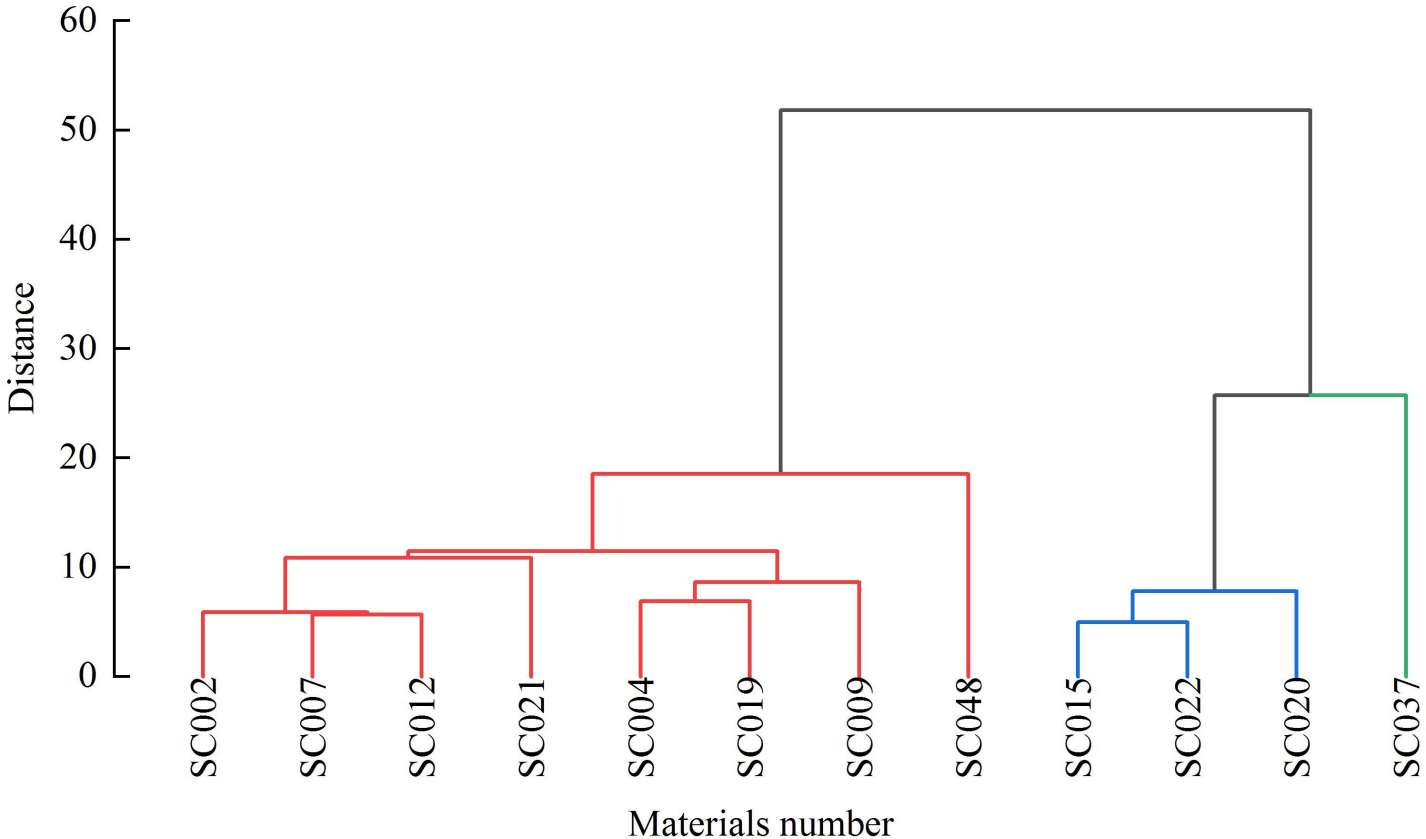
Quality cluster analysis of twelve germplasm resources of the genus *Allium*.

Cluster analysis was conducted on 15 quality indices of the twelve germplasm resources of the genus *Allium*, and a cluster tree chart was established. At a Euclidean distance of 20, the twelve germplasm resources could be divided into three major categories. The first category consisted of eight materials, including SC048, SC019, SC009, SC004, SC007, SC022, SC012, and SC021, that ranked 1-8 comprehensively and accounted for 66.67%. The second category consisted of three materials (SC002, SC020, and SC015) with a comprehensive ranking of 9-11 that accounted for 25%. SC037 with a comprehensive ranking of 12 was grouped separately into the third category and accounted for 8.33% (**Figure 6**).

## 4 Discussion

The phenotype is determined by the genotype and living environment, and quantitative characteristics are easily influenced by the external environment (Langstroff et al., 2022). During plant growth, the phenological phase reflects the comprehensive impact of climate on the plant and is an important indicator of if the plant can adapt to the region and grow normally (Stuble et al., 2021). Through two consecutive years of observation of the phenological period of twelve germplasm resources of the genus *Allium* from Tibet, it was observed that three twelve germplasm resources were unable to normally bolt and flower, likely due to the observation that some plants of the genus *Allium* collected from Tibet grew under severe agroclimatic conditions such as harsh, cold winters, and high altitudes and thus evolved to be non-bolting. The bolting, flowering, and seed harvest phase of the other nine plants remained highly consistent within species, whereas there were significant differences between species, and this is consistent with the results for wild plants of the genus *Allium* in Inner Mongolia (Yang et al., 2013). We investigated the quantitative characteristics of twelve germplasm resources of the genus *Allium* and observed that single plant weight, flowering stalk length, and flowering stalk diameter were the most diverse; however, the variation coefficient of plant height was the smallest, and which was determined by their genotype.

*Allium* species are revered worldwide as spices with sharp and tang odors and pungent spicy taste (Singh et al., 2018). Pyruvic acid is closely related to the pungent taste of the genus *Allium* and can be used to determine the pungency of the genus *Allium* (Gîtin et al., 2014). In this study, there was a significant variation in pyruvic acid content among different germplasm resources of the genus *Allium*, thus result to the significant differences in spicy taste. Besides, volatile compounds are important factors that affect the flavor of the genus *Allium*. Different volatile compounds cause different flavor sensations. Eight types of volatile compounds were identified in the twelve germplasm resources included aldehydes, organosulfur compounds, ketones, alcohols, terpenoids, alkanes, and furans. The majority of germplasm resources possessed the highest proportions of aldehydes and organosulfur compounds, but there were certain differences between the different *Allium* species. These results are in line with the previous findings that the volatile compounds identified in the genus *Allium* primarily include sulfides, aldehydes, ketones, alcohols, hydrocarbons, furans, and heterocyclic compounds (Yin et al., 2019; Xie et al., 2022; Liu et al., 2022a). OSC determines the unique aroma and taste of the genus *Allium*, making them a potential symbol of the genus *Allium*, but the type and content of OSC vary depending on the species of *Allium* plants, and this results in clearly distinguish in flavors and fragrances (Baky et al., 2022). In this study, different degrees of OSCs were detected in all twelve germplasm resources of the genus *Allium*, and which was the most diverse compound among all volatiles. The dimethyl trisulfide content accounted for the largest proportion in the species of *Allium tuberosum* Rottl. ex Spreng and *Allium hookeri* Thwaites. Aldehyde was primarily volatile compounds, secondly OSCs in *Allium przewalskianum* species, which exhibited intense the aroma of green grass, and 3,5-diethyl-1,2,4-trichlorothecyclopentane and 2,5-dimethylthiophene was primary and unique component of OSCs. Similar results were obtained in wild plants of the genus *Allium* in the Qinghai-Tibet Plateau (Guan et al., 2020), leeks (Yabuki et al., 2010; Zhang et al., 2012) and *Allium przewalskianum*, but differently, the primary and unique component of OSCs in *Allium przewalskianum* are methylpropyl sulfide and dipropyl sulfide (Lang et al., 2018).

Plants of the genus *Allium* are rich in amino acids that play important roles in nutrition, medicine, and sensory value (He et al., 2018). Except for Cys, a total of 16 free amino acids including eight EAA and eight NEAA were detected in twelve germplasm resources examined in this study. Similarly, Liu et al. (2022b) also reported that Chinese chive contains almost Cys, thus indicating that leek and *A. przewalskianum* are extremely lack of Cys. Studies have demonstrated that different amino acids cause different flavors in plants of the genus *Allium*. Glu and Asp provide umami flavor, whereas Ser, Gly, Ala, Pro, and Gln contribute to a sweet taste (Abdelrahman et al., 2020). The twelve germplasm resources examined in this study exhibited significant interspecies differences in amino acid composition and content, the biggest disparity in the total amino acid content was nearly three-fold, and this led to significant flavor differences among the different plants of the genus *Allium* in this study. And almost all germplasm resources possessed the highest content of Pro, and followed by Glu. This is similar to the results of the highest content of Glu in wild Leek in Tibet (Wang et al., 2017). It is speculated that Pro is related to plant resistance. To adapt to cold and high-altitude environments, these plants of the genus *Allium* in Tibet have evolved strong resistance to abiotic stress.

Mineral elements in vegetables are the main sources of essential elements for the human body (Loppi et al., 2021). The essential element content in twelve germplasm resources of the genus *Allium* basically meets the need of the human body for macro- and micro-elements. As the basic macroelement, C content was the highest, and followed by K, and Fe was the highest microelement, the contents of the other mineral elements except C varied greatly among the different materials. This is consistent with the results for garlic, onion, and leek (Czech et al., 2022; Gao et al., 2022).

Comprehensive evaluation such as correlation analysis, principal component analysis, and cluster analysis is useful method to evaluate the quality of germplasm resources (Liu et al., 2023). In the present study, through comprehensive evaluation of fifteen quality traits, it suggested that SC048 possessed advantage in comprehensive quality, and this was followed by SC019 and SC009. Similar method has been reported on pears, raspberries, and other fruits (Yazdanpour et al., 2018; Han et al., 2022). Besides, it also found some germplasm resources with high content of mineral elements, amino acids or OSCs alone, which can be further cultivated and used for quality breeding of the genus *Allium*.

## 5 Conclusion

Though observing the biological characteristics, the germplasm resources of SC015, SC019, and SC048 were unable to bolt and bloom normally, whereas the remaining nine germplasm resources performed well. The twelve germplasm resources of the genus *Allium* varied in basic nutritional quality, content of amino acid, OSCs and essential elements. Comprehensive evaluation of the 15 quality indices showed that SC048 possessed advantage in comprehensive quality, followed by SC019 and SC009. In briefly, combined with biological performance and comprehensive evaluation of quality, SC009 is the excellent germplasm resource that were suitable for growth and capable of reproduction with good quality. The findings of this study are practically significant for utilization and high-quality breeding of the genus *Allium*.

## Data availability statement

The original contributions presented in the study are included in the article/**Supplementary Material**. Further inquiries can be directed to the corresponding author.

## Author contributions

HW: Investigation, Writing – original draft. HY: Investigation, Writing – original draft. XY: Investigation, Writing – original draft. YX: Investigation, Writing – original draft. YB: Investigation, Writing – original draft. QD: Investigation, Writing – original draft. LL: Investigation, Writing – original draft. WT: Investigation, Writing – original draft. MY: Investigation, Writing – original draft. LW: Investigation, Writing – original draft. ZH: Investigation, Writing – original draft. BS: Investigation, Writing – original draft. HL: Investigation, Writing – original draft. YT: Conceptualization, Writing – review & editing.
