# Supplementary material for: Biological features and quality comprehensive analysis of twelve germplasm resources of the genus Allium from Tibet
Source: Front Plant Sci. 2024 Aug 6;15:1393402. doi: 10.3389/fpls.2024.1393402 (PMC11333262; doi:10.3389/fpls.2024.1393402)
Supplement: Supplementary file 1 [file Table_1.docx]

Table S1 Comparison of quantitative characters of twelve germplasm resources of the genus *Allium*

| Characters | Material number | | | | | | | | | | | |
| --- | --- | --- | --- | --- | --- | --- | --- | --- | --- | --- | --- | --- |
|  | SC002 | SC004 | SC007 | SC009 | SC012 | SC015 | SC019 | SC020 | SC021 | SC022 | SC037 | SC048 |
| Plant height (cm) | 27.45±2.41f | 36.50±2.17cd | 38.73±1.90c | 28.08±1.65f | 38.68±2.05c | 51.18±3.82a | 29.89±2.92ef | 46.10±2.55b | 33.30±2.37de | 34.80±2.79cd | 32.49±2.21de | 52.90±2.48a |
| Tiller number | 3.67±0.58f | 2.00±0.00g | 3.00±0.00f | 5.75±0.50de | 20.00±1.83a | 5.22±0.44de | 4.75±0.50e | 10.67±0.58c | 6.25±0.50d | 13.11±0.60b | 3.67±0.58f | 3.20±0.45f |
| Leaves number per plant | 4.33±0.58d | 4.25±0.50e | 6.00±1.00cd | 2.75±0.50f | 8.60±0.55b | 6.20±0.42cd | 4.33±0.58e | 6.86±0.69c | 5.75±0.50d | 9.89±0.60a | 4.43±1.13e | 4.80±0.45e |
| Fresh weight per plant (g) | 1.26±0.45f | 2.17±0.16f | 2.30±0.44f | 1.07±0.19f | 16.36±1.59d | 50.89±5.05a | 1.80±0.61f | 8.75±1.19e | 17.86±1.57c | 2.61±0.23f | 2.88±0.28f | 39.05±6.82b |
| Leave length (cm) | 20.43±2.80f | 26.47±2.92e | 30.37±1.76d | 20.02±1.40f | 35.13±2.35c | 47.98±2.55a | 18.60±2.81f | 34.80±2.99c | 27.02±1.85de | 28.94±0.93de | 27.03±1.96de | 42.06±2.27b |
| Leave width (mm) | 1.45±0.11d | 1.63±0.20d | 2.31±0.17d | 1.57±0.26d | 10.75±0.56a | 9.60±0.69ab | 1.31±0.13d | 7.80±0.87bc | 11.19±0.94a | 6.95±0.61c | 1.71±0.15d | 9.87±0.81ab |
| Pseudostem length(cm) | 7.02±1.08cd | 4.50±0.70ef | 8.43±1.17bc | 7.89±1.13bc | 3.18±0.42f | 9.20±0.30b | 8.90±0.76b | 11.55±1.04a | 5.71±0.79de | 5.86±1.22de | 5.93±0.86de | 10.84±1.02a |
| Pseudostem (mm) | 5.07±0.38h | 6.62±1.08efg | 7.05±0.91ef | 3.20±0.18i | 9.25±0.93c | 23.49±1.05b | 2.12±0.57i | 7.61±0.57de | 6.27±0.55fgh | 8.71±0.12cd | 5.66±0.38gh | 16.24±1.69b |
| Flowering stalk length  (cm) | 10.41±0.75e | 23.87±1.35c | 27.57±1.85c | 23.00±1.46c | 24.22±2.62c | / | / | 57.91±6.09a | 25.51±3.44c | 46.14±3.54b | 15.20±0.79d | / |
| Flowering stalk diameter (mm) | 1.36±0.17f | 1.96±0.38d | 1.62±0.19def | 1.49±0.12ef | 2.68±0.28c | / | / | 3.94±0.44b | 3.74±0.14b | 4.37±0.44a | 1.87±0.19de | / |
| Bud length(mm) | 12.52±1.18d | 11.99±1.47d | 13.33±1.44d | 11.97±0.46d | 22.31±1.76a | / | / | 21.43±1.52a | 16.52±1.84c | 19.04±1.87b | 11.18±1.92d | / |
| Bud width(mm) | 3.82±0.41d | 5.81±0.95c | 5.66±0.45c | 5.73±0.95c | 7.00±0.61b | / | / | 9.23±0.83a | 8.89±0.31a | 6.16±0.48bc | 5.40±1.03c | / |
| Ball-flower diameter (mm) | 26.57±2.16f | 43.78±3.04b | 33.67±2.52cde | 37.16±2.25c | 32.57±2.72de | / | / | 54.20±1.89a | 35.81±4.85cd | 54.21±3.25a | 29.47±2.37ef | / |

“/” represents not detected. Data represents means (±SD) of thirty independent replicates. Different lowercase letters indicate significant differences within a column (LSD *P*≤0.05).
