# Supplementary material for: Biological features and quality comprehensive analysis of twelve germplasm resources of the genus Allium from Tibet
Source: Front Plant Sci. 2024 Aug 6;15:1393402. doi: 10.3389/fpls.2024.1393402 (PMC11333262; doi:10.3389/fpls.2024.1393402)
Supplement: Supplementary file 2 [file Table_2.docx]

Table S2 Comparison of volatile compounds of twelve germplasm resources of the genus *Allium*

| Type | CAS | Compound | Relative content（%） | | | | | | | | | | | |
| --- | --- | --- | --- | --- | --- | --- | --- | --- | --- | --- | --- | --- | --- | --- |
|  |  |  | SC002 | SC004 | SC007 | SC009 | SC012 | SC015 | SC019 | SC020 | SC021 | SC022 | SC037 | SC048 |
| Aldehydes | 75-07-0 | Acetaldehyde | 2.27 | 2.57 | 3.41 | 2.67 | 0.87 | / | 5.59 | 1.55 | 2.02 | 2.46 | 6.03 | / |
|  | 123-38-6 | [Propanal](https://www.chemsrc.com/en/cas/123-73-9_165164.html) | 2.74 | 3.98 | 2.36 | 3.42 | 0.24 | 1.40 | 4.89 | 0.09 | / | / | 3.45 | / |
|  | 123-73-9 | [Crotonaldehyde](https://www.chemsrc.com/en/cas/3034-50-2_1194710.html) | 4.77 | 3.90 | 3.71 | 4.76 | 2.71 | 0.99 | 3.95 | 2.28 | 2.27 | 5.82 | 1.73 | 1.30 |
|  | 3034-50-2 | [1H-Imidazole-4-carbaldehyde](https://www.chemsrc.com/en/cas/497-03-0_1101535.html) | / | / | 0.18 | 0.22 | / | / | / | / | / | / | 0.28 | / |
|  | 497-03-0 | [trans-2-Methyl-2-butenal](https://www.chemsrc.com/en/cas/1576-87-0_894831.html) | 11.80 | 11.74 | 7.37 | 11.43 | 0.64 | 3.03 | 11.34 | 0.34 | 0.34 | 1.99 | / | 2.05 |
|  | 1576-87-0 | [(E)-2-Pentenal](https://www.chemsrc.com/en/cas/66-25-1_406582.html) | 0.57 | 0.63 | 0.71 | 1.09 | 0.16 | / | 0.78 | / | / | / | / | / |
|  | 66-25-1 | [Hexanal](https://www.chemsrc.com/en/cas/623-36-9_86466.html) | 3.20 | 2.10 | 1.16 | 2.48 | 0.33 | 2.71 | 1.95 | 0.20 | 0.20 | 1.38 | 1.72 | / |
|  | 623-36-9 | [2-Methyl-2-pentenal](https://www.chemsrc.com/en/cas/505-57-7_687814.html) | 3.94 | 6.30 | 5.01 | 6.98 | / | 0.97 | 10.40 | / | / | / | 6.89 | 0.83 |
|  | 505-57-7 | [2-hexen-1-al](https://www.chemsrc.com/en/cas/123-72-8_1085302.html) | 18.09 | 12.96 | 9.44 | 11.73 | 1.42 | 11.42 | 9.08 | 1.04 | 1.03 | 6.95 | 12.21 | 2.78 |
|  | 123-72-8 | [Butyraldehyde](https://www.chemsrc.com/en/cas/100-52-7_946834.html) | / | / | / | / | / | 0.33 | / | / | / | / | / | / |
|  | 100-52-7 | [Benzaldehyde](https://www.chemsrc.com/en/cas/645-62-5_2454.html) | / | 0.30 | 0.15 | 0.35 | 1.41 | 0.55 | / | 1.04 | 1.04 | 0.26 | / | 0.50 |
|  | 645-62-5 | 2-Ethyl-2-hexenal | / | 1.33 | / | / | / | / | / | / | / | / | / | / |
|  | 004313-03-5 | trans, trans-2,4-Heptadienal | / | / | / | / | / | 1.48 | / | / | / | / | / | / |
|  | 1000154-24-0 | Cyclopentanecarboxaldehyde, 2-methyl-3-methylene- | 2.92 | / | 0.88 | / | / | / | 1.26 | / | / | / | / | / |
|  | 2548-87-0 | (E)-2-Octenal | 0.82 | / | / | / | / | / | / | / | / | 0.81 | / | / |
|  | 124-19-6 | Nonanal | 4.12 | 3.20 | 1.71 | 1.38 | / | 4.99 | 2.38 | / | / | 3.38 | 2.32 | 4.75 |
|  | 60784-31-8 | [(Z)-2-nonen-1-al](https://www.chemsrc.com/en/cas/432-25-7_270417.html) | 1.23 | / | / | / | / | / | / | / | / | 1.16 | / | / |
|  | 432-25-7 | [β-Cyclocitral](https://www.chemsrc.com/en/cas/74-93-1_195668.html) | / | / | 0.72 | / | / | 1.81 | / | / | / | / | / | / |
| Alcohols | 74-93-1 | [Methyl mercaptan](https://www.chemsrc.com/en/cas/75-15-0_336228.html) | / | / | / | / | / | / | / | / | / | 0.67 | / | / |
|  | 75-15-0 | [Carbon disulphide](https://www.chemsrc.com/en/cas/1072-43-1_1137962.html) | 0.79 | 0.42 | 0.21 | 0.32 | / | 0.42 | 0.70 | / | / | / | 0.74 | / |
|  | 1072-43-1 | [2-Methylthiirane](https://www.chemsrc.com/en/cas/624-92-0_1028175.html) | / | / | 0.19 | 1.52 | / | / | / | / | / | / | / | / |
|  | 624-92-0 | [Dimethyl disulfide](https://www.chemsrc.com/en/cas/638-02-8_430950.html) | / | / | / | / | 0.91 | / | / | 1.00 | 1.00 | 2.48 | / | / |
|  | 638-02-8 | [2,5-Dimethylthiophene](https://www.chemsrc.com/en/cas/505-23-7_242918.html) | 8.44 | 8.09 | 7.69 | 9.40 | 0.25 | 7.50 | 8.28 | 0.62 | 0.61 | / | 9.96 | 10.23 |
|  | 505-23-7 | 1,3-Dithiane | / | / | / | / | 7.52 | / | 0.35 | 8.55 | 8.52 | 5.48 | 3.01 | 0.73 |
| Organosulfurs | 631-67-4 | [N, N-Dimethylthioacetamide](https://www.chemsrc.com/en/cas/3658-80-8_80208.html) | / | / | / | / | 0.18 | / | / | / | / | / | / | / |
|  | 3658-80-8 | [Dimethyl trisulfide](https://www.chemsrc.com/en/cas/26555-40-8_897812.html) | / | / | / | / | 16.70 | / | / | 16.95 | 16.89 | 24.01 | 3.54 | / |
|  | 26555-40-8 | [Methoxycarbonylsulfenyl chloride](https://www.chemsrc.com/en/cas/2949-92-0_752803.html) | / | / | / | / | 0.21 | / | / | 0.18 | / | / | / | / |
|  | 2949-92-0 | [Methyl Methanethiosulfonate](https://www.chemsrc.com/en/cas/17249-80-8_25982.html) | / | / | / | / | / | / | / | / | / | 2.96 | / | / |
|  | 17249-80-8 | [3-Chlorothiophene](https://www.chemsrc.com/en/cas/2179-57-9_952389.html) | / | / | / | / | 0.25 | / | / | / | / | 0.65 | / | / |
|  | 2179-57-9 | [Diallyl disulfide](https://www.chemsrc.com/en/cas/7210-63-1_1614257.html) | / | / | / | / | 2.34 | / | / | 1.95 | 1.94 | 0.70 | / | / |
|  | 7210-63-1 | [4-Methylthiothiophen-2(5H)-one](https://www.chemsrc.com/en/cas/74804-37-8_623326.html) | / | / | / | / | 2.18 | / | / | 2.01 | 2.00 | / | / | / |
|  | 74804-37-8 | [1,4,6-Oxadiazocane-5-thione](https://www.chemsrc.com/en/cas/629-19-6_195789.html) | / | / | / | / | 2.99 | / | / | 3.20 | 3.19 | 1.30 | / | / |
|  | 629-19-6 | [Propyl disulfide](https://www.chemsrc.com/en/cas/1438-16-0_404008.html) | / | / | / | / | / | 0.63 | / | / | / | / | / | / |
|  | 1438-16-0 | [N-Aminorhodanine](https://www.chemsrc.com/en/cas/13105-10-7_1669832.html) | / | / | / | / | / | 1.21 | / | / | / | / | 0.54 | / |
|  | 13105-10-7 | [1,2-Bis(ethylthio)ethene](https://www.chemsrc.com/en/cas/557-22-2_32165.html) | / | / | / | / | 0.33 | / | / | / | / | / | / | / |
|  | 557-22-2 | 1,2-Dithiolane | / | / | / | / | / | / | / | 0.42 | 0.41 | / | / | / |
|  | 42474-44-2 | 2,3,5-Trithiahexane | / | / | / | / | / | / | / | 0.77 | 0.77 | / | / | / |
|  | 2373-51-5 | Chloromethyl Methyl Sulfide | / | / | / | / | 2.53 | / | / | / | / | / | / | / |
|  | 556-64-9 | Methyl thiocyanate | / | / | / | / | 25.84 | / | / | 25.09 | 24.99 | 8.52 | / | / |
|  | 997-49-9 | Trimethyl trithioborate | / | / | / | / | / | / | / | / | / | 0.66 | / | / |
|  | 52195-40-1 | (Z)-2-Thia-3-pentene | / | / | / | / | 4.15 | 1.40 | 1.37 | 5.23 | 5.12 | / | 1.83 | 3.03 |
|  | 31207-14-4 | α-bromomethylthiirane | / | / | / | / | / | / | / | / | / | / | 4.40 | / |
|  | 16890-71-4 | N-Methyldithioxamide | / | / | / | / | / | / | / | 0.18 | / | / | / | / |
|  | 62488-52-2 | 3-ethenyl-3,6-dihydrodithiine | / | / | / | / | 0.56 | / | / | 0.25 | 0.25 | / | / | / |
|  | 5756-24-1 | Dimethyltetrasulfane | / | / | / | / | 2.95 | / | / | 2.51 | 2.50 | 0.94 | 0.35 | / |
|  | 105643-80-9 | 2-prop-2-enylsulfanylacetonitrile | / | / | / | / | 1.58 | / | / | 1.20 | 1.19 | / | / | / |
|  | 26246-29-7 | 2-Thiazoline, 2-amino-4-imino- | / | / | / | / | 1.28 | / | / | 1.14 | 1.14 | / | / | / |
|  | 33922-80-4 | [1-Propene,1,1'-thiobis-](https://www.chemsrc.com/en/cas/38348-25-3_658051.html) | / | / | / | / | / | / | / | 2.02 | 2.01 | / | / | / |
|  | 38348-25-3 | [3,5-Diethyl-1,2,4-trithiolane](https://www.chemsrc.com/en/cas/592-88-1_1028176.html) | 4.64 | 5.04 | 4.43 | 4.39 | / | / | 4.00 | / | / | / | 4.14 | / |
|  | 592-88-1 | [Allyl sulfide](https://www.chemsrc.com/en/cas/1516-70-7_28723.html) | / | 0.74 | 1.09 | 0.94 | 1.95 | 5.77 | / | / | / | / | 0.77 | 9.07 |
|  | 1516-70-7 | [Methane sulfonyl azide](https://www.chemsrc.com/en/cas/2758-18-1_1109005.html) | / | / | / | / | / | / | / | 0.33 | / | / | / | / |
| Ketones | 2758-18-1 | 3-Methyl-2-cyclopenten-1-one | / | 0.24 | / | / | / | / | 0.40 | / | / | / | / | / |
|  | 585-25-1 | [2,3-Octadione](https://www.chemsrc.com/en/cas/110-93-0_509694.html) | 1.54 | 1.06 | 0.87 | 0.80 | / | / | 0.55 | / | / | / | 1.39 | / |
|  | 110-93-0 | [6-Methylhept-5-en-2-one](https://www.chemsrc.com/en/cas/5090-16-4_717268.html) | / | / | 0.85 | 0.86 | / | / | 0.69 | / | / | / | 1.19 | 1.47 |
|  | 5090-16-4 | [3-Hepten-2-one, 5-methyl-](https://www.chemsrc.com/en/cas/1604-28-0_279144.html) | 3.80 | / | / | / | / | / | / | / | / | 1.11 | 0.77 | / |
|  | 1604-28-0 | [6-Methyl-3,5-heptadiene-2-one](https://www.chemsrc.com/en/cas/546-80-5_251604.html) | / | / | / | 1.60 | / | / | / | / | / | 0.81 | / | / |
|  | 546-80-5 | [α-Thujone](https://www.chemsrc.com/en/cas/69393-15-3_985249.html) | / | / | / | / | 3.26 | / | / | 5.21 | 5.19 | / | 1.52 | 4.29 |
|  | 69393-15-3 | [1-(1H-Imidazol-4-yl)-1-pentanone](https://www.chemsrc.com/en/cas/108-96-3_332883.html) | 0.75 | / | 15.29 | / | / | / | / | / | / | / | / | / |
|  | 108-96-3 | 4(1H)-Pyridinone | / | / | / | / | 0.38 | / | / | / | / | / | / | / |
|  | 105480-28-2 | Ethanone,2,2,2-trifluoro-1-(1H-imidazol-5-yl)- | 0.49 | / | / | / | / | / | / | 0.42 | 0.42 | / | 0.64 | / |
|  | 14901-07-6 | [Beta-Ionone](https://www.chemsrc.com/en/cas/18409-17-1_151977.html) | / | / | 0.30 | 0.34 | / | 2.21 | / | / | / | / | / | 1.28 |
| Alcohols | 18409-17-1 | [Trans-2-Octen-1-ol](https://www.chemsrc.com/en/cas/104-76-7_1101393.html) | / | 1.61 | 2.16 | 1.31 | / | / | / | / | / | / | / | 3.67 |
|  | 104-76-7 | [2-Ethylhexanol](https://www.chemsrc.com/en/cas/100-51-6_895867.html) | / | 0.80 | 0.41 | / | / | 1.21 | / | / | / | 0.75 | / | / |
|  | 100-51-6 | [Benzyl alcohol](https://www.chemsrc.com/en/cas/507-70-0_600406.html) | / | / | / | / | / | 0.65 | / | / | / | / | / | 1.61/ |
|  | 507-70-0 | [Borneol](https://www.chemsrc.com/en/cas/4430-77-7_955830.html) | / | / | 8.31 | / | 1.98 | / | / | 2.76 | 2.75 | / | / | 3.22 |
|  | 4430-77-7 | [Pyrido[2,3-d] pyridazine-5,8-diol](https://www.chemsrc.com/en/cas/1115-11-3_453124.html) | / | / | 0.19 | / | / | / | / | / | / | / | / | / |
| Terpenes | 1115-11-3 | [Tiglic aldehyde](https://www.chemsrc.com/en/cas/100-42-5_28937.html) | / | / | / | / | / | / | / | / | / | / | 5.90 | / |
|  | 100-42-5 | [Styrene](https://www.chemsrc.com/en/cas/3168-90-9_411372.html) | 11.59 | 10.95 | 7.66 | 5.98 | 4.16 | 22.06 | 15.00 | 3.50 | 3.49 | 12.59 | 11.05 | 18.41 |
|  | 3168-90-9 | [1-(2-methyl-1- cyclopentenyl) ethanone](https://www.chemsrc.com/en/cas/74421-05-9_141913.html) | / | / | 0.77 | / | / | / | / | / | / | / | / | / |
|  | 74421-05-9 | [2,4-Dimethyl-2,4-heptadiene](https://www.chemsrc.com/en/cas/918-86-5_712000.html) | / | / | / | / | / | / | / | / | / | / | 0.57 | / |
|  | 918-86-5 | 3-methylpenta-1,4-dien-3-ol | / | / | 0.79 | / | / | / | / | / | / | / | / | / |
|  | 1000194-22-7 | [1,2-Dimethoxy-ethene](https://www.chemsrc.com/en/cas/5256-65-5_635082.html) | / | / | 0.87 | / | / | / | / | / | / | 1.50 | / | / |
|  | 5256-65-5 | p-Mentha-2-ene | / | / | 0.41 | 0.46 | / | / | / | / | / | / | / | / |
| Alkanes | 75-21-8 | [Ethylene oxide](https://www.chemsrc.com/en/cas/503-30-0_405283.html) | / | / | / | / | / | 1.96 | / | / | / | / | / | / |
|  | 503-30-0 | Oxetane | / | / | / | / | / | / | / | / | / | / | / | 1.25 |
|  | 100688-50-4 | [1-chloro-3-[2-(2-methoxyethoxy) ethoxy]-](https://www.chemsrc.com/en/cas/10471-14-4_912815.html) | / | / | / | / | / | / | 1.45 | / | / | / | / | / |
|  | 10471-14-4 | [Acetaldehyde ethyl methyl acetal](https://www.chemsrc.com/en/cas/2408-37-9_437312.html) | / | / | / | / | / | 2.07 | / | / | / | / | / | / |
|  | 2408-37-9 | [2,2,6-trimethylcyclohexanone](https://www.chemsrc.com/en/cas/541-02-6_512376.html) | / | / | / | / | / | 0.66 | / | / | / | / | / | / |
|  | 541-02-6 | [Decamethylcyclopentasiloxane](https://www.chemsrc.com/en/cas/53366-51-1_997084.html) | / | / | 1.56 | 2.27 | / | / | / | / | / | / | / | / |
|  | 53366-51-1 | (3-Methylbutylidene) cyclopentane | / | 0.18 | / | / | / | / | / | / | / | / | / | / |
|  | 2679-87-0 | [Butane, 2-ethoxy-](https://www.chemsrc.com/en/cas/107-50-6_42789.html) | / | / | / | / | / | / | / | 1.18 | 1.17 | / | / | / |
|  | 107-50-6 | [Tetradecamethyl Cycloheptasiloxane](https://www.chemsrc.com/en/cas/96-47-9_1153686.html) | 0.89 | / | / | / | / | / | / | / | / | / | / | / |
| Furans | 96-47-9 | [2-Methyltetrahydrofuran](https://www.chemsrc.com/en/cas/3194-17-0_196930.html) | 3.00 | 4.49 | 2.96 | 2.64 | 1.80 | 13.99 | 4.71 | 2.28 | 2.27 | 4.92 | 6.78 | 13.46 |
|  | 3194-17-0 | [2-Valerylfuran](https://www.chemsrc.com/en/cas/1192-62-7_952383.html) | / | / | / | 6.42 | / | / | / | / | / | / | / | / |
|  | 1192-62-7 | [1-(Furan-2-yl) ethanone](https://www.chemsrc.com/en/cas/1703-52-2_139368.html) | / | 3.86 | / | / | / | / | / | / | / | 0.44 | / | / |
|  | 1703-52-2 | [2-ethyl-5-methyl furan](https://www.chemsrc.com/en/cas/6909-30-4_524678.html) | / | / | / | 4.13 | / | / | 0.45 | / | / | / | / | / |
| Others | 6909-30-4 | [(+)-Trans-limonene1, 2-epoxide](https://www.chemsrc.com/en/cas/321-28-8_952291.html) | / | 1.01 | / | / | / | / | / | / | / | / | / | / |
|  | 321-28-8 | 2-Fluoroanisole | / | / | 1.82 | 1.12 | / | / | / | / | / | / | / | / |
|  | 2461-15-6 | [2-Ethylhexyl glycidyl ether](https://www.chemsrc.com/en/cas/42392-30-3_399999.html) | / | / | / | / | / | / | / | 0.48 | 0.48 | / | / | / |
|  | 42392-30-3 | [Cyclobutanecarboxylic acid cyclobutyl ester](https://www.chemsrc.com/en/cas/504-88-1_1029441.html) | / | / | / | / | / | / | / | / | / | 0.94 | / | / |
|  | 504-88-1 | [3-Nitropropanoic acid](https://www.chemsrc.com/en/cas/700-13-0_895852.html) | / | / | / | / | / | 0.31 | / | / | / | / | / | / |
|  | 700-13-0 | [2,3,5-Trimethyl-1,4-benzenediol](https://www.chemsrc.com/en/cas/61985-25-9_131488.html) | / | / | / | / | / | / | / | / | / | / | / | 2.01 |
|  | 61985-25-9 | [1-(1H-Imidazol-4-yl) ethanone hydrochloride (1:1)](https://www.chemsrc.com/en/cas/36947-68-9_1193042.html) | / | / | / | / | / | 0.85 | / | / | / | / | / | / |
|  | 36947-68-9 | [2-Isopropylimidazole](https://www.chemsrc.com/en/cas/109-12-6_1092285.html) | / | / | / | 0.13 | / | / | / | / | / | / | / | / |
|  | 109-12-6 | [2-Aminopyrimidine](https://www.chemsrc.com/en/cas/607-91-0_755226.html) | / | 2.23 | / | / | / | / | / | / | / | / | / | / |
|  | 607-91-0 | Myristicin | / | / | / | / | 0.68 | / | / | / | / | / | / | / |

"/" indicates the data here is not detected.
